# Diethyl (*E*)-2,3-bis­[(*E*)-(2-methyl-2-phenyl­hydrazin-1-yl­idene)meth­yl]but-2-enedioate

**DOI:** 10.1107/S1600536814011970

**Published:** 2014-05-31

**Authors:** Peng Liu, Libin Yuan, Xiuqing Song, Hong Yan

**Affiliations:** a College of Life Science and Bio-engineering, Beijing University of Technology, Pingleyuan Street No. 100, Chaoyang District, Beijing 100124, People’s Republic of China

## Abstract

The complete mol­ecule of the title compound, C_24_H_28_N_4_O_4_, is generated by crystallographic inversion symmetry. The ethyl side chain is disordered over two sets of sites in a 0.57 (4):0.43 (4) ratio. The dihedral angles between the methyl­idene group and the phenyl ring and ester side chain (major conformation) are 7.61 (8) and 86.95 (8)°, respectively. In the crystal, mol­ecules are linked *via* C—H⋯O hydrogen bonds, forming corrugated sheets lying parallel to (010).

## Related literature   

For background to this class of compound, see: Aumann *et al.* (1987 ▶). For studies of related mol­ecules, see: Mandal & Basak (2009 ▶); Woerlee *et al.* (1984 ▶).
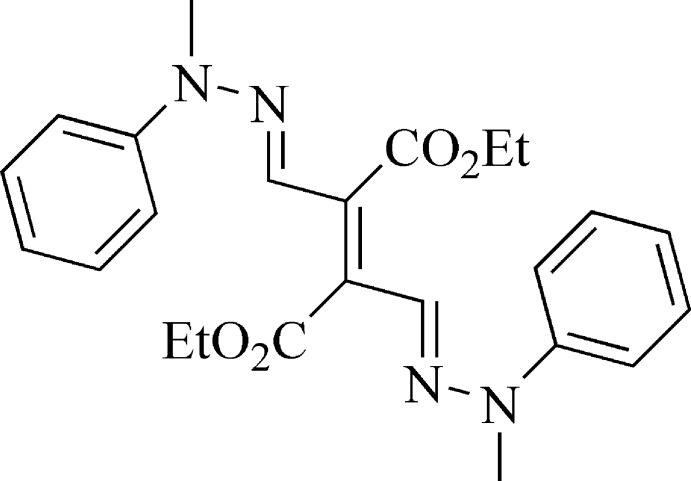


## Experimental   

### 

#### Crystal data  

C_24_H_28_N_4_O_4_
*M* *_r_* = 436.50
Orthorhombic, 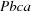

*a* = 15.922 (3) Å
*b* = 8.0335 (16) Å
*c* = 18.655 (4) Å
*V* = 2386.2 (8) Å^3^
*Z* = 4
Mo *K*α radiation
μ = 0.08 mm^−1^
*T* = 113 K
0.20 × 0.10 × 0.08 mm

#### Data collection  

Rigaku Saturn CCD diffractometer
Absorption correction: multi-scan (*CrystalClear*; Rigaku/MSC, 2005 ▶) *T*_min_ = 0.983, *T*_max_ = 0.993
14849 measured reflections
2093 independent reflections
1925 reflections with *I* > 2σ(*I*)
*R*_int_ = 0.034

#### Refinement  

*R*[*F* ^2^ > 2σ(*F* ^2^)] = 0.038
*wR*(*F* ^2^) = 0.099
*S* = 1.08
2093 reflections
168 parameters
40 restraints
H-atom parameters constrained
Δρ_max_ = 0.18 e Å^−3^
Δρ_min_ = −0.16 e Å^−3^

### 

Data collection: *CrystalClear* (Rigaku/MSC, 2005 ▶); cell refinement: *CrystalClear*; data reduction: *CrystalClear*; program(s) used to solve structure: *SHELXS97* (Sheldrick, 2008 ▶); program(s) used to refine structure: *SHELXL97* (Sheldrick, 2008 ▶); molecular graphics: *SHELXTL* (Sheldrick, 2008 ▶); software used to prepare material for publication: *SHELXTL*.

## Supplementary Material

Crystal structure: contains datablock(s) I, New_Global_Publ_Block. DOI: 10.1107/S1600536814011970/hb7226sup1.cif

Structure factors: contains datablock(s) I. DOI: 10.1107/S1600536814011970/hb7226Isup2.hkl

Supporting information file. DOI: 10.1107/S1600536814011970/hb7226Isup3.cml

CCDC reference: 1004694

Additional supporting information:  crystallographic information; 3D view; checkCIF report

## Figures and Tables

**Table 1:** Hydrogen-bond geometry (Å, °)

*D*—H⋯*A*	*D*—H	H⋯*A*	*D*⋯*A*	*D*—H⋯*A*
C4—H4*A*⋯O1^i^	0.95	2.47	3.3749 (19)	160

Symmetry code: (i) 
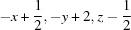
.

**Fig. 1.**
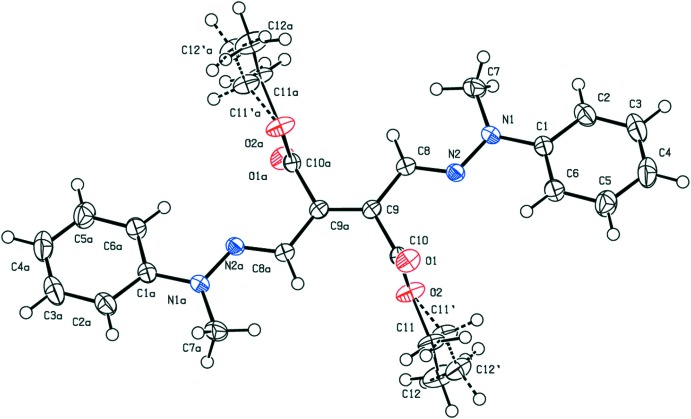
Ellipsoid plot with displacement ellipsoids drawn at the 50% probability level.

## supplementary crystallographic information

### 1. Experimental

Ethyl 3-ethoxy-2-nitroacrylate (10.6 mmol) and hydrazine (10.6 mmol) were stirred in ethanol, and the solution was heated briefly to boiling. A yellow solid was collected by filtration after the mixture was left to stand for 24 h. The product was recrystallized from dichloromethane and petroleum ether as red blocks in 31% yield (m.p. 160° C).

### Crystal data

**Table:**

C_24_H_28_N_4_O_4_	*D*_x_ = 1.215 Mg m^−^^3^
*M**_r_* = 436.50	Mo *K*α radiation, λ = 0.71073 Å
Orthorhombic, *P**b**c**a*	Cell parameters from 6374 reflections
*a* = 15.922 (3) Å	θ = 2.2–27.9°
*b* = 8.0335 (16) Å	µ = 0.08 mm^−^^1^
*c* = 18.655 (4) Å	*T* = 113 K
*V* = 2386.2 (8) Å^3^	Block, red
*Z* = 4	0.20 × 0.10 × 0.08 mm
*F*(000) = 928	

### Data collection

**Table:**

Rigaku Saturn CCD diffractometer	2093 independent reflections
Radiation source: rotating anode	1925 reflections with *I* > 2σ(*I*)
Multilayer monochromator	*R*_int_ = 0.034
Detector resolution: 7.31 pixels mm^-1^	θ_max_ = 25.0°, θ_min_ = 2.5°
ω and φ scans	*h* = −15→18
Absorption correction: multi-scan (*CrystalClear*; Rigaku/MSC, 2005)	*k* = −9→9
*T*_min_ = 0.983, *T*_max_ = 0.993	*l* = −22→22
14849 measured reflections	

### Refinement

**Table:**

Refinement on *F*^2^	Secondary atom site location: difference Fourier map
Least-squares matrix: full	Hydrogen site location: inferred from neighbouring sites
*R*[*F*^2^ > 2σ(*F*^2^)] = 0.038	H-atom parameters constrained
*wR*(*F*^2^) = 0.099	*w* = 1/[σ^2^(*F*_o_^2^) + (0.0585*P*)^2^ + 0.373*P*] where *P* = (*F*_o_^2^ + 2*F*_c_^2^)/3
*S* = 1.08	(Δ/σ)_max_ = 0.001
2093 reflections	Δρ_max_ = 0.18 e Å^−^^3^
168 parameters	Δρ_min_ = −0.16 e Å^−^^3^
40 restraints	Extinction correction: *SHELXL97* (Sheldrick, 2008), Fc^*^=kFc[1+0.001xFc^2^λ^3^/sin(2θ)]^-1/4^
Primary atom site location: structure-invariant direct methods	Extinction coefficient: 0.063 (4)

### Special details

**Table:**

Geometry. All e.s.d.'s (except the e.s.d. in the dihedral angle between two l.s. planes) are estimated using the full covariance matrix. The cell e.s.d.'s are taken into account individually in the estimation of e.s.d.'s in distances, angles and torsion angles; correlations between e.s.d.'s in cell parameters are only used when they are defined by crystal symmetry. An approximate (isotropic) treatment of cell e.s.d.'s is used for estimating e.s.d.'s involving l.s. planes.
Refinement. Refinement of *F*^2^ against ALL reflections. The weighted *R*-factor *wR* and goodness of fit *S* are based on *F*^2^, conventional *R*-factors *R* are based on *F*, with *F* set to zero for negative *F*^2^. The threshold expression of *F*^2^ > σ(*F*^2^) is used only for calculating *R*-factors(gt) *etc*. and is not relevant to the choice of reflections for refinement. *R*-factors based on *F*^2^ are statistically about twice as large as those based on *F*, and *R*- factors based on ALL data will be even larger.

### Fractional atomic coordinates and isotropic or equivalent isotropic displacement parameters (Å^2^)

**Table:**

	*x*	*y*	*z*	*U*_iso_*/*U*_eq_	Occ. (<1)
O1	0.17130 (5)	0.95435 (11)	0.47833 (5)	0.0331 (3)	
O2	0.08700 (5)	0.74716 (11)	0.44282 (5)	0.0363 (3)	
N1	0.03970 (6)	1.17222 (12)	0.29214 (5)	0.0269 (3)	
N2	0.05365 (6)	1.08830 (12)	0.35347 (5)	0.0240 (3)	
C1	0.10279 (7)	1.15535 (14)	0.23935 (6)	0.0258 (3)	
C2	0.09073 (9)	1.21349 (17)	0.16961 (7)	0.0365 (3)	
H2A	0.0404	1.2705	0.1574	0.044*	
C3	0.15209 (11)	1.18797 (19)	0.11824 (7)	0.0468 (4)	
H3A	0.1438	1.2298	0.0711	0.056*	
C4	0.22471 (10)	1.1034 (2)	0.13400 (8)	0.0484 (4)	
H4A	0.2658	1.0845	0.0980	0.058*	
C5	0.23704 (9)	1.04608 (18)	0.20314 (7)	0.0416 (4)	
H5A	0.2870	0.9872	0.2145	0.050*	
C6	0.17769 (8)	1.07337 (16)	0.25580 (7)	0.0315 (3)	
H6A	0.1878	1.0363	0.3034	0.038*	
C7	−0.03664 (8)	1.26664 (17)	0.28254 (7)	0.0373 (3)	
H7A	−0.0408	1.3515	0.3201	0.056*	
H7B	−0.0851	1.1917	0.2855	0.056*	
H7C	−0.0358	1.3208	0.2355	0.056*	
C8	0.00195 (7)	1.09756 (14)	0.40658 (6)	0.0243 (3)	
H8A	−0.0473	1.1640	0.4044	0.029*	
C9	0.02301 (7)	1.00148 (13)	0.46959 (6)	0.0229 (3)	
C10	0.10276 (7)	0.90180 (14)	0.46439 (6)	0.0235 (3)	
C11	0.1581 (6)	0.6302 (13)	0.4421 (8)	0.0421 (19)	0.57 (4)
H11A	0.1836	0.6218	0.4904	0.051*	0.57 (4)
H11B	0.2017	0.6679	0.4079	0.051*	0.57 (4)
C12	0.1231 (7)	0.4658 (13)	0.4194 (9)	0.052 (2)	0.57 (4)
H12A	0.1689	0.3854	0.4140	0.078*	0.57 (4)
H12B	0.0938	0.4784	0.3735	0.078*	0.57 (4)
H12C	0.0835	0.4258	0.4558	0.078*	0.57 (4)
C11'	0.1622 (7)	0.6478 (17)	0.4289 (12)	0.045 (3)	0.43 (4)
H11C	0.1926	0.6258	0.4743	0.054*	0.43 (4)
H11D	0.2003	0.7077	0.3959	0.054*	0.43 (4)
C12'	0.1334 (9)	0.4862 (17)	0.3956 (10)	0.048 (2)	0.43 (4)
H12D	0.1802	0.4070	0.3948	0.071*	0.43 (4)
H12E	0.1143	0.5070	0.3465	0.071*	0.43 (4)
H12F	0.0871	0.4399	0.4238	0.071*	0.43 (4)

### Atomic displacement parameters (Å^2^)

**Table:**

	*U*^11^	*U*^22^	*U*^33^	*U*^12^	*U*^13^	*U*^23^
O1	0.0242 (5)	0.0348 (5)	0.0403 (5)	−0.0016 (4)	−0.0039 (4)	−0.0055 (4)
O2	0.0245 (5)	0.0250 (5)	0.0593 (6)	0.0043 (4)	−0.0072 (4)	−0.0100 (4)
N1	0.0295 (6)	0.0269 (5)	0.0241 (5)	0.0043 (4)	−0.0007 (4)	0.0049 (4)
N2	0.0276 (6)	0.0224 (5)	0.0221 (5)	0.0000 (4)	−0.0013 (4)	0.0010 (4)
C1	0.0306 (7)	0.0229 (6)	0.0238 (6)	−0.0058 (5)	0.0006 (5)	−0.0013 (4)
C2	0.0445 (8)	0.0366 (7)	0.0283 (7)	−0.0022 (6)	−0.0028 (6)	0.0055 (5)
C3	0.0619 (10)	0.0532 (9)	0.0254 (7)	−0.0081 (8)	0.0061 (7)	0.0062 (6)
C4	0.0516 (10)	0.0571 (9)	0.0366 (7)	−0.0069 (7)	0.0177 (7)	−0.0026 (7)
C5	0.0345 (8)	0.0477 (8)	0.0427 (8)	−0.0009 (6)	0.0099 (6)	−0.0005 (6)
C6	0.0303 (7)	0.0356 (7)	0.0285 (6)	−0.0015 (5)	0.0016 (5)	0.0024 (5)
C7	0.0341 (8)	0.0414 (8)	0.0366 (7)	0.0111 (6)	−0.0008 (6)	0.0104 (6)
C8	0.0237 (6)	0.0228 (6)	0.0263 (6)	0.0015 (5)	−0.0008 (5)	−0.0013 (4)
C9	0.0231 (6)	0.0210 (6)	0.0245 (6)	−0.0014 (5)	−0.0012 (5)	−0.0031 (4)
C10	0.0259 (7)	0.0249 (6)	0.0196 (6)	−0.0001 (5)	−0.0003 (5)	0.0000 (4)
C11	0.027 (2)	0.028 (2)	0.071 (4)	0.0120 (19)	−0.006 (2)	−0.007 (2)
C12	0.045 (3)	0.029 (3)	0.083 (5)	0.010 (2)	−0.012 (3)	−0.015 (3)
C11'	0.032 (3)	0.029 (3)	0.073 (6)	0.008 (2)	−0.005 (3)	−0.016 (3)
C12'	0.037 (3)	0.032 (3)	0.074 (5)	0.006 (2)	0.004 (4)	−0.007 (3)

### Geometric parameters (Å, º)

**Table:**

O1—C10	1.1987 (14)	C7—H7A	0.9800
O2—C10	1.3298 (15)	C7—H7B	0.9800
O2—C11'	1.462 (8)	C7—H7C	0.9800
O2—C11	1.472 (6)	C8—C9	1.4458 (16)
N1—N2	1.3463 (13)	C8—H8A	0.9500
N1—C1	1.4133 (15)	C9—C9^i^	1.351 (2)
N1—C7	1.4440 (16)	C9—C10	1.5042 (16)
N2—C8	1.2902 (14)	C11—C12	1.495 (7)
C1—C2	1.3956 (17)	C11—H11A	0.9900
C1—C6	1.3964 (18)	C11—H11B	0.9900
C2—C3	1.384 (2)	C12—H12A	0.9800
C2—H2A	0.9500	C12—H12B	0.9800
C3—C4	1.373 (2)	C12—H12C	0.9800
C3—H3A	0.9500	C11'—C12'	1.511 (8)
C4—C5	1.383 (2)	C11'—H11C	0.9900
C4—H4A	0.9500	C11'—H11D	0.9900
C5—C6	1.3807 (18)	C12'—H12D	0.9800
C5—H5A	0.9500	C12'—H12E	0.9800
C6—H6A	0.9500	C12'—H12F	0.9800
			
C10—O2—C11'	114.1 (6)	H7A—C7—H7C	109.5
C10—O2—C11	117.0 (5)	H7B—C7—H7C	109.5
C11'—O2—C11	11.4 (12)	N2—C8—C9	116.46 (10)
N2—N1—C1	115.27 (9)	N2—C8—H8A	121.8
N2—N1—C7	120.49 (10)	C9—C8—H8A	121.8
C1—N1—C7	124.21 (10)	C9^i^—C9—C8	124.51 (14)
C8—N2—N1	121.22 (10)	C9^i^—C9—C10	120.18 (13)
C2—C1—C6	118.71 (11)	C8—C9—C10	115.31 (10)
C2—C1—N1	121.31 (11)	O1—C10—O2	124.51 (11)
C6—C1—N1	119.94 (10)	O1—C10—C9	124.54 (11)
C3—C2—C1	119.92 (13)	O2—C10—C9	110.95 (10)
C3—C2—H2A	120.0	O2—C11—C12	106.2 (7)
C1—C2—H2A	120.0	O2—C11—H11A	110.5
C4—C3—C2	121.30 (13)	C12—C11—H11A	110.5
C4—C3—H3A	119.3	O2—C11—H11B	110.5
C2—C3—H3A	119.3	C12—C11—H11B	110.5
C3—C4—C5	118.93 (13)	H11A—C11—H11B	108.7
C3—C4—H4A	120.5	O2—C11'—C12'	107.1 (10)
C5—C4—H4A	120.5	O2—C11'—H11C	110.3
C6—C5—C4	120.90 (14)	C12'—C11'—H11C	110.3
C6—C5—H5A	119.6	O2—C11'—H11D	110.3
C4—C5—H5A	119.6	C12'—C11'—H11D	110.3
C5—C6—C1	120.20 (12)	H11C—C11'—H11D	108.6
C5—C6—H6A	119.9	C11'—C12'—H12D	109.5
C1—C6—H6A	119.9	C11'—C12'—H12E	109.5
N1—C7—H7A	109.5	H12D—C12'—H12E	109.5
N1—C7—H7B	109.5	C11'—C12'—H12F	109.5
H7A—C7—H7B	109.5	H12D—C12'—H12F	109.5
N1—C7—H7C	109.5	H12E—C12'—H12F	109.5
			
C1—N1—N2—C8	177.93 (10)	N2—C8—C9—C9^i^	178.85 (14)
C7—N1—N2—C8	−4.05 (16)	N2—C8—C9—C10	−0.46 (15)
N2—N1—C1—C2	169.89 (11)	C11'—O2—C10—O1	−6.8 (10)
C7—N1—C1—C2	−8.05 (18)	C11—O2—C10—O1	5.4 (7)
N2—N1—C1—C6	−7.57 (15)	C11'—O2—C10—C9	173.7 (10)
C7—N1—C1—C6	174.49 (12)	C11—O2—C10—C9	−174.1 (7)
C6—C1—C2—C3	0.60 (19)	C9^i^—C9—C10—O1	−92.64 (17)
N1—C1—C2—C3	−176.88 (12)	C8—C9—C10—O1	86.70 (14)
C1—C2—C3—C4	1.2 (2)	C9^i^—C9—C10—O2	86.85 (16)
C2—C3—C4—C5	−1.4 (2)	C8—C9—C10—O2	−93.81 (12)
C3—C4—C5—C6	−0.2 (2)	C10—O2—C11—C12	177.4 (7)
C4—C5—C6—C1	1.9 (2)	C11'—O2—C11—C12	−104 (6)
C2—C1—C6—C5	−2.13 (18)	C10—O2—C11'—C12'	−172.3 (7)
N1—C1—C6—C5	175.39 (11)	C11—O2—C11'—C12'	81 (5)
N1—N2—C8—C9	179.66 (10)		

Symmetry code: (i) −*x*, −*y*+2, −*z*+1.

### Hydrogen-bond geometry (Å, º)

**Table:**

*D*—H···*A*	*D*—H	H···*A*	*D*···*A*	*D*—H···*A*
C4—H4*A*···O1^ii^	0.95	2.47	3.3749 (19)	160

Symmetry code: (ii) −*x*+1/2, −*y*+2, *z*−1/2.
